# Predicting in-hospital mortality after transcatheter aortic valve replacement using administrative data and machine learning

**DOI:** 10.1038/s41598-023-37358-9

**Published:** 2023-06-24

**Authors:** Theyab Alhwiti, Summer Aldrugh, Fadel M. Megahed

**Affiliations:** 1 School of Management, Clark University, Worcester, MA USA; 2 Division of Cardiovascular Medicine, Department of Medicine, University of Massachusetts Medical School, Worcester, MA USA; 3 Farmer School of Business, Miami University, Oxford, OH USA

**Keywords:** Interventional cardiology, Information technology, Outcomes research

## Abstract

Transcatheter aortic valve replacement (TAVR) is the gold standard treatment for patients with symptomatic aortic stenosis. The utility of existing risk prediction tools for in-hospital mortality post-TAVR is limited due to two major factors: (a) the predictive accuracy of these tools is insufficient when only preoperative variables are incorporated, and (b) their efficacy is also compromised when solely postoperative variables are employed, subsequently constraining their application in preoperative decision support. This study examined whether statistical/machine learning models trained with solely preoperative information encoded in the administrative National Inpatient Sample database could accurately predict in-hospital outcomes (death/survival) post-TAVR. Fifteen popular binary classification methods were used to model in-hospital survival/death. These methods were evaluated using multiple classification metrics, including the area under the receiver operating characteristic curve (AUC). By analyzing 54,739 TAVRs, the top five classification models had an AUC ≥ 0.80 for two sampling scenarios: random, consistent with previous studies, and time-based, which assessed whether the models could be deployed without frequent retraining. Given the minimal practical differences in the predictive accuracies of the top five models, the L2 regularized logistic regression model is recommended as the best overall model since it is computationally efficient and easy to interpret.

## Introduction

Administrative/claims data maintained by government payers and private insurers have been increasingly used for monitoring and improving health care performance^1–4^. For example, the US National Inpatient Sample (NIS) from the Healthcare Cost and Utilization Project (HCUP) is an observational, anonymized database in which the unit of analysis is the discharge record^5^. The NIS captures several demographic variables, admission date, discharge date and status, primary and secondary International Classification of Diseases (ICD) diagnoses, procedures, length of stay, etc^4,5^. In the context of cardiac surgical outcomes, the NIS has been used to (a) identify surgical adverse outcomes and improve patient safety^2,6^; (b) assess the efficacy/cost of surgical outcomes for specific patient populations^7^; and (c) predict in-hospital death using statistical and/or machine learning models^1,3^.

While the scope and size of administrative data (e.g., the NIS database) “affords wonderful research latitude”^8^, such data have inherent limitations^9^ since they were originally collected for billing purposes. In the context of predictive studies, the following limitations are the most pertinent: (a) the lack of clinical data; (b) the surveillance bias phenomenon of “the more you look, the more you find,” which can make the study of certain diagnoses/complications invalid^10^; and (c) the volume of data (i.e., big data) can make *p* values for statistical significance frequently much less than the typical cutoff of 0.05^8^. Despite these limitations, we utilized the NIS database in this paper since it is publicly available, incorporates multiple geographic regions, and continues to play an important role in health service research^9^.

The overarching goal of this study was to examine whether the preoperative information encoded in the administrative NIS database could accurately predict in-hospital death/survival after transcatheter aortic valve replacement (TAVR), which is the “gold standard treatment for patients with severe symptomatic aortic stenosis”^3^ and has been recently expanded to include low-surgical risk patients^11^. To achieve this goal, we examined the utility of both statistical and machine learning models for predicting in-hospital death post-TAVR procedures based on the discussion in^3,12–14^. We utilized the performance assessment score of^1^, which assessed the performance of predictive models using “receiver operating characteristic (ROC) curve scores measuring discrimination (< 0.7 = poor, 0.7–0.8 = reasonable, > 0.8 = good)”. Hence, we examined whether an area under the ROC curve (AUC) value of > 0.8 could be achieved using only the preoperative information. Thus, this study can be considered a follow-up/extension to^3^, which showed that in-hospital deaths post-TAVR procedures can be predicted using both pre- and postoperative NIS variables. Furthermore, we examined whether such models could be deployed in practice without frequent retraining by investigating the differences in predictive performance when the training and holdout samples were stratified by time order.

## Results

### Baseline characteristics

Table 1 presents the baseline characteristics of our dataset (*n* = 54,739). There were 1113 (2.03%) in-hospital patient deaths before discharge. Additionally, we found that the patients who died in the hospital were more likely to be older; female; to have a history of fluid and electrolyte disorders, implantable cardioverter defibrillator (ICD), peripheral vascular disease, cardiac arrhythmias, chronic kidney disease, anemia, pulmonary circulation disorder, atrial fibrillation and flutter, chronic pulmonary disease, liver disease, and coagulopathy; and to be admitted to an urban nonteaching hospital in the south. In addition, these patients were less likely to have a history of cancer, carotid artery disease, dyslipidemia, valvular disease, smoking, coronary artery bypass graft (CABG), coronary artery disease (CAD), myocardial infarction (MI), permanent pacemaker (PPM), percutaneous coronary intervention (PCI), and transient ischemic attack (TIA). A total of 43,791 (80%) and 10,948 (20%) patients were randomly assigned to the development and validation cohorts, respectively. Patient characteristics were similar between the development and validation cohorts. A total of 39,820 (74.5%) and 13,982 (25.5%) patients were split based on the time from 2012 to 2018 and 2019, respectively. The characteristics were similar between the two sets (see Supplementary Table S1 for the full list and question 2).

**Table 1:** Demographic, hospital, and comorbidity characteristics of TAVR patients.

	Overall		Survived		Deceased		*p* value	Training dataset		Test dataset		*p* value
Demographic characteristics	N = 54,739		53,626		1113			43,791		10,948		
Age (years)	79.65	± 8.5	79.62	± 8.5	81	± 8.9	< 0.001	80	± 8.5	80	± 8.6	0.622
Sex (female)	25,229	(46.1)	24,629	(45.9)	600	(53.9)	< 0.001	20,215	(46.2)	5,014	(45.8)	0.494
Smoker	20,793	(38)	20,537	(38.3)	256	(23)	< 0.001	16,690	(38.1)	4103	(37.5)	0.22
Dyslipidemia	38,654	(70.6)	38,102	(71.1)	552	(49.6)	< 0.001	30,954	(70.7)	7700	(70.3)	0.468
Atrial fibrillation and flutter	22,215	(40.6)	21,678	(40.4)	537	(48.3)	< 0.001	17,733	(40.5)	4482	(40.9)	0.397
Carotid artery disease	3611	(6.6)	3563	(6.6)	48	(4.3)	0.002	2889	(6.6)	722	(6.6)	0.993
Known CAD	38,673	(70.7)	37,959	(70.8)	714	(64.2)	< 0.001	30,972	(70.7)	7701	(70.3)	0.429
Prior CABG	9714	(17.8)	9569	(17.8)	145	(13)	< 0.001	7830	(17.9)	1884	(17.2)	0.099
Prior ICD	1528	(2.8)	1503	(2.8)	25	(2.3)	0.265	1182	(2.7)	346	(3.2)	0.01
Prior MI	6980	(12.8)	6861	(12.8)	119	(10.7)	0.037	5599	(12.8)	1381	(12.6)	0.63
Prior PCI	11,975	(21.9)	11,828	(22.1)	147	(13.2)	< 0.001	9566	(21.8)	2409	(22)	0.718
Prior PPM	5351	(9.8)	5278	(9.8)	73	(6.6)	< 0.001	4315	(9.9)	1036	(9.5)	0.218
Prior TIA/stroke	76	(13.9)	7482	(14)	122	(11)	0.004	6071	(13.9)	1533	(14)	0.707
Elixhauser comorbidity												
Anemia	5084	(9.3)	4957	(9.2)	127	(11.4)	0.017	4087	(9.3)	997	(9.1)	0.466
Cancer	1469	(2.7)	1440	(2.7)	29	(2.6)	0.871	1176	(2.7)	293	(2.7)	0.958
Cardiac arrhythmias	29,382	(53.7)	28,626	(53.4)	756	(67.9)	0.0001	23,498	(53.7)	5884	(53.7)	0.872
Chronic kidney disease	11,978	(21.9)	11,613	(21.7)	365	(32.8)	< 0.001	9603	(21.9)	2375	(21.7)	0.594
Chronic pulmonary disease	17,549	(32.1)	17,113	(31.9)	436	(39.2)	< 0.001	14,037	(32.1)	3512	(32.1)	0.961
Coagulopathy	7706	(14.1)	7353	(13.7)	535	(31.7)	< 0.001	6144	(14)	1562	(14.3)	0.523
Depression	4441	(8.1)	4378	(8.2)	63	(5.7)	0.002	3535	(8.1)	906	(8.3)	0.486
Diabetes mellitus	20,257	(37)	19,936	(37.2)	321	(28.8)	< 0.001	16,258	(37.1)	3999	(36.5)	0.246
Fluid and electrolyte disorders	9206	(16.8)	8624	(16.1)	582	(52.3)	< 0.001	7397	(16.9)	1809	(16.5)	0.357
Heart failure	40,541	(74.1)	39,653	(73.9)	888	(79.8)	< 0.001	32,427	(74.1)	8114	(74.1)	0.89
Hypertension	48,328	(88.3)	47,482	(88.5)	846	(76)	< 0.001	38,711	(88.4)	9617	(84.8)	0.105
Liver disease	1888	(3.5)	1712	(3.2)	176	(15.8)	< 0.001	1504	(3.4)	384	(3.5)	0.708
Peripheral vascular disease	13,220	(24.2)	12,859	(24)	361	(32.4)	< 0.001	10,486	(24)	2734	(25)	0.025
Pulmonary circulation disorder	7001	(12.8)	6777	(12.6)	224	(20.1)	< 0.001	5607	(12.8)	1394	(12.7)	0.842
Valvular disease	53,831	(98.3)	52,760	(98.4)	1071	(96.2)	< 0.001	43,048	(98.3)	10,783	(98.5)	0.165
Family history of CAD	3778	(6.9)	3735	(7)	43	(3.9)	< 0.001	3028	(6.9)	750	(6.9)	0.813
Hospital location												
Rural	536	(1)	524	(1)	12	(1.1)	0.926	414	(1)	122	(1.1)	0.237
Urban nonteaching hospital	4880	(8.9)	4779	(8.9)	101	(90.7)		3920	(9)	960	(8.8)	
Urban teaching hospital	49,323	(90.1)	48,323	(90.1)	1000	(89.9)		39,457	(90.1)	9866	(90.1)	
Hospital region												
Northeast	13,432	(24.6)	13,193	(24.6)	239	(21.5)	< 0.001	10,813	(24.7)	2619	(23.9)	0.146
Midwest	11,765	(21.5)	11,512	(21.5)	253	(22.7)		9415	(21.5)	2350	(21.5)	
South	18,742	(34.2)	18,304	(34.1)	438	(39.4)		14,998	(34.3)	3744	(34.2)	
West	10,800	(19.7)	10,617	(19.8)	183	(16.4)		8565	(19.6)	2235	(20.4)	
Other	879	(1.6)	866	(1.6)	13	(1.2)		667	(1.5)	153	(1.4)	
Transapical TAVR	45,078	(82.4)	44,308	(82.6)	770	(69.2)	< 0.001	36,010	(82.2)	9068	(82.8)	0.143
Endovascular TAVR	9681	(17.7)	9335	(17.4)	346	(31.1)	< 0.001	7781	(17.8)	1880	(17.2)	0.143

### ML classifiers’ performance for in-hospital mortality prediction based on preoperative information

**Figure 1:**
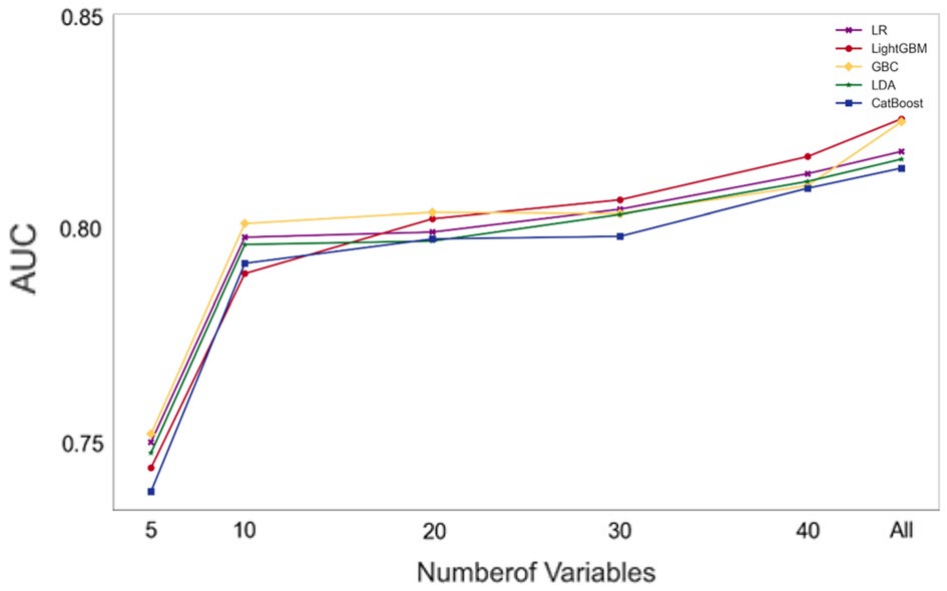
Performance of the top five models by the number of input variables for research question 1. A similar figure for question 2 is presented in the supplementary materials.

Table 2 presents the baseline results from the dummy model along with the testing/holdout results of our top five classification models with varying (5/10/20/30/40/all) feature sets and different holdout datasets for our first (preoperative predictors and random sampling) and second (preoperative predictors and time-based sampling) research questions. Irrespective of the model, the AUC, balanced accuracy, sensitivity, precision, and F1 score improved as more features were used for prediction. Figure 1 depicts the improvement in the AUC as more features were made available to the model.

**Table 2:** Predictive performance of the top 5 ML models in the test/holdout datasets. The abbreviations refer to: a baseline dummy classifier that predicts outcomes based on the most frequent class, logistic regression implementing an L2 penalty (hereafter abbreviated as LR), Light Gradient Boosting Machine (LightGBM), Gradient Boosting Classifier (GBC), and the CatBoost Classifier.

	ML model	# Input features	AUC	Balanced accuracy	Sensitivity	Specificity	Precision	NPV	F1
Question 1	Dummy	0	0.5	0.5	0	1	0	0.979	0
	LR	All	0.817	0.743	0.715	0.771	0.063	0.992	0.116
		40	0.813	0.741	0.711	0.771	0.062	0.992	0.114
		30	0.804	0.735	0.702	0.767	0.060	0.992	0.111
		20	0.799	0.711	0.658	0.763	0.056	0.991	0.103
		10	0.798	0.716	0.667	0.765	0.057	0.991	0.105
		5	0.750	0.679	0.566	0.792	0.055	0.988	0.100
	LightGBM	All	0.825	0.740	0.803	0.677	0.052	0.993	0.097
		40	0.817	0.730	0.785	0.675	0.049	0.993	0.092
		30	0.807	0.717	0.768	0.667	0.047	0.993	0.089
		20	0.802	0.717	0.781	0.653	0.048	0.994	0.090
		10	0.789	0.705	0.746	0.665	0.045	0.992	0.085
		5	0.744	0.678	0.750	0.605	0.039	0.985	0.075
	GBC	All	0.824	0.719	0.627	0.810	0.064	0.992	0.116
		40	0.810	0.719	0.627	0.810	0.064	0.992	0.117
		30	0.803	0.731	0.689	0.773	0.063	0.992	0.115
		20	0.804	0.719	0.675	0.763	0.057	0.999	0.105
		10	0.801	0.716	0.662	0.769	0.056	0.991	0.104
		5	0.752	0.681	0.570	0.791	0.057	0.988	0.104
	LDA	All	0.816	0.743	0.702	0.785	0.065	0.992	0.120
		40	0.811	0.742	0.697	0.786	0.065	0.992	0.118
		30	0.803	0.732	0.684	0.780	0.062	0.991	0.114
		20	0.797	0.716	0.654	0.779	0.059	0.991	0.109
		10	0.796	0.704	0.627	0.781	0.057	0.990	0.105
		5	0.748	0.677	0.526	0.828	0.061	0.988	0.110
	CatBoost	All	0.814	0.724	0.654	0.795	0.066	0.983	0.120
		40	0.809	0.728	0.662	0.794	0.065	0.983	0.118
		30	0.798	0.716	0.654	0.778	0.057	0.982	0.105
		20	0.798	0.704	0.640	0.767	0.055	0.984	0.101
		10	0.792	0.701	0.640	0.761	0.053	0.984	0.099
		5	0.739	0.679	0.575	0.784	0.043	0.986	0.079
Question 2	Dummy	0	0.5	0.5	0	1	0	0.987	0
	LR	All	0.808	0.732	0.619	0.846	0.049	0.994	0.090
		40	0.812	0.730	0.614	0.845	0.048	0.994	0.089
		30	0.808	0.716	0.574	0.859	0.049	0.994	0.091
		20	0.802	0.717	0.574	0.861	0.050	0.994	0.092
		10	0.796	0.722	0.614	0.831	0.044	0.994	0.083
		5	0.781	0.709	0.580	0.839	0.044	0.994	0.082
	LightGBM	All	0.810	0.717	0.676	0.757	0.034	0.995	0.064
		40	0.808	0.722	0.671	0.773	0.034	0.994	0.064
		30	0.810	0.730	0.688	0.773	0.036	0.995	0.068
		20	0.805	0.728	0.671	0.786	0.036	0.995	0.069
		10	0.796	0.718	0.705	0.731	0.032	0.995	0.062
		5	0.777	0.706	0.739	0.674	0.027	0.995	0.052
	GBC	All	0.810	0.715	0.585	0.846	0.049	0.994	0.091
		40	0.793	0.706	0.563	0.850	0.051	0.994	0.094
		30	0.808	0.722	0.591	0.854	0.047	0.994	0.087
		20	0.792	0.725	0.585	0.865	0.050	0.993	0.091
		10	0.784	0.724	0.608	0.840	0.043	0.994	0.079
		5	0.775	0.710	0.585	0.834	0.040	0.993	0.074
	LDA	All	0.809	0.713	0.585	0.842	0.050	0.994	0.093
		40	0.813	0.725	0.591	0.858	0.051	0.994	0.093
		30	0.808	0.712	0.557	0.868	0.049	0.994	0.091
		20	0.803	0.714	0.557	0.871	0.052	0.994	0.095
		10	0.797	0.726	0.608	0.844	0.047	0.994	0.088
		5	0.782	0.704	0.557	0.851	0.045	0.993	0.084
	CatBoost	All	0.800	0.720	0.574	0.866	0.053	0.990	0.097
		40	0.799	0.728	0.597	0.860	0.053	0.991	0.097
		30	0.799	0.725	0.580	0.871	0.082	0.990	0.125
		20	0.802	0.713	0.568	0.859	0.064	0.991	0.106
		10	0.795	0.709	0.591	0.827	0.038	0.991	0.070
		5	0.761	0.701	0.557	0.846	0.034	0.992	0.064

**Figure 2:**
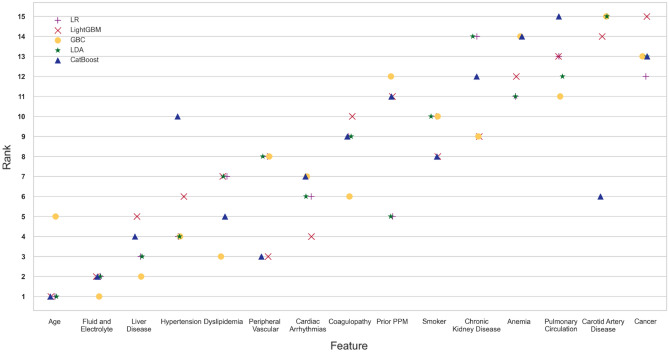
Ranked feature importance for each of the top five models based on the mean ranking for research question 1. A similar figure for question 2 is presented in the supplementary materials.

The importance of the predictors varied primarily with the classification model used. Figure 2 (Supplementary Table S2) shows the ranked importance of the features for each model for research question 1. Overall, age was the most important feature for all models, except for the GBC model, which had fluid and electrolyte disorders as its top feature (the second most important feature for the other four models). The presence of liver disease, hypertension, peripheral vascular disease, dyslipidemia, cardiac arrhythmias, and smoking were among the features consistently picked as important by the models.

## Discussion

Herein, we proposed using publicly available administrative data to predict in-hospital mortality post-TAVR using 15 classification models. Comparing the results of our top 5 classification models with the baseline dummy classification benchmark, five observations were made. First, our classification metrics generally improved as more features were made available to the model, with modest improvements after the top 20–30 features. This indicates that the use of chi-square tests to select the top variables was suitable for our dataset since it did not contradict the results obtained when the boosting-based algorithms and ridge regression were used with all features. Second, all five models were able to provide “good” predictive results per^1^ definition of an AUC ≥ 0.80 for both the random and time-based sampling scenarios (Table 2). These AUC values generally corresponded when ≥ 30 top features were available for the model. Third, it is important to note that the holdout results were consistent with our cross-validation results (see Supplementary Table S3), indicating no evidence of overfitting. Fourth, the LR model presented the best overall predictive performance for our two research questions since its AUC, balanced accuracy, sensitivity, precision, and F1 score were among the highest values observed across all models. For example, it had the highest balanced accuracy of 0.743 and 0.732 (i.e., the arithmetic mean of sensitivity and specificity). The other metrics were all significantly higher than those of the dummy classifier and practically equivalent to those of the best performing model for each metric. We recommend the LR model since it is computationally efficient and is the easiest to interpret among the five top models. Fifth, the precision and F1 scores for all the models were relatively low (approximately 10%); however, this was expected given the highly imbalanced nature of our dataset. Note that the dummy classifier returned a value of 0 with its default setting, and it would have returned a value approximately equal to our survival percentage of ~ 2% if the stratified input was selected^15^.

Our feature importance in Fig. 2 is somewhat similar to the top features reported by^3^. However, four of their top five features (acute kidney injury, cardiogenic shock, cardiac arrest, and sepsis) were not available for selection by our models since they were all postsurgery complications. Only fluid and electrolyte disorders, which were their third most important feature, were available for selection by our model.

While several studies have examined the use of statistical and/or machine learning models for TAVR prediction^3,16–21^, few studies have examined in-hospital mortality^3,16,17^. In these papers, the reported AUC scores were (a) 0.66 based on 9 preoperative variables in^16^, (b) 0.92 based on a combination of pre- and postoperative predictors in^3^, and (c) not reported in^17^ as logistic regression was used to compute the odds ratio for predictors of mortality in the adjusted analysis of patients who underwent TAVR with end-stage renal disease. Our original sample size of 54,739 was much larger than the 10,891, 20,540, and 6,836 patients used in the other studies. Furthermore, our AUC value of > 0.81 was significantly larger than the 0.66 reported in^16^. While it is less than the 0.92 reported in^3^, the differences are attributed to not including postoperative predictors; our top models had AUC values of 0.91–0.93 when postoperative predictors were available during model building (Supplementary Table S4). We computed the performance of the models with postoperative data only to support our previous statement and to show that the models’ predictive performance could reach the values reported in^3^ (given that our dataset included low-risk patients who were not in their dataset, and the authors did not make their code available).

This study demonstrated the feasibility of using solely the preoperative information available in administrative data to accurately predict in-hospital mortality post-TAVR. Our study represents the first report in which “good” predictive performance^1^ could be achieved using solely preoperative, administrative predictors. Our results mark a substantial improvement, an increase in the AUC of approximately 0.15, over the results of^16^, while addressing the main limitation in^3^, i.e., “the inability to restrict variables pre-procedure versus post-procedure, which provides a dynamic nature to the NIS TAVR score.” Specifically, we showed that by removing any of the variables that could have occurred “post-procedure”, a model could still have good predictive performance and quantify the impact of not including such variables on the predictive performance (a decrease in the AUC from 0.91–0.93 to 0.81). Our approach is consistent with the recommendation in^13^ who stated that “for decision support algorithms to be implemented in clinical practice, we would expect them to be accurate and pertinent at the time a decision is taken.” Furthermore, we showed that the developed models may not need to be retrained often, since the predictive performance for the 2019 holdout dataset in question 2 was similar to that obtained from random sampling in our first research question. To our knowledge, this is the first study in which deployment-related questions were assessed with respect to TAVR operations. In our estimation, our time-based sampling approach presents a methodological approach to “subject [decision support models for clinical practice] to the test of time” per the recommendation of^13^.

Despite the examination of a relatively large number of machine learning models, our study showed that the predictive performance of an L2 regularized logistic regression model was equivalent to the results obtained using more complex machine learning models. This is also consistent with the results obtained in^13^ using postoperative predictors. This confirmatory result can accelerate the use/deployment of logistic regression as a preoperative risk scoring tool for TAVR procedures. In our estimation, the use of a logistic-type model would be preferable in medical practice for four main reasons. First, it is an explainable model, i.e., when the coefficients are exponentiated, we can capture the change in odds when one predictor is increased by one unit, holding other predictors constant. Hence, this follows the recommendations of^22^, who recommended the use of interpretable models for high-stakes decisions, and^13^, who stated that “clinicians and patients should also be provided enough information to understand the process that led to the decision.” Second, statistical tests for variable significance and model goodness-of-fit analyses can be performed to provide additional insight about the model. Third, due to its interpretability and good predictive performance, it is meaningful to not only look at the dichotomized prediction but also to extract the underlying survival/death probability from the model. We anticipate that reporting a preprocedural survival probability would inform clinical pathway determination and provide a structured, data-driven risk adjustment of expected outcomes. Fourth, regularized logistic regression can be performed using multiple software programs that are currently used in medical settings^23^.

While this study utilized only administrative, preoperative variables in model building, it is interesting to note that our reported predictive accuracy metrics were, at a minimum, similar to (if not exceeding) a large amount of the TAVR risk assessment literature in which clinical data were utilized (often for a slightly longer 30-day prediction period). In 2015, the American College of Cardiology (ACC) and the Society of Transthoracic Surgeons (STS) developed an in-hospital mortality risk score based on STS/ACC transcatheter valve therapy (TVT) registry data^16^. This risk score took into account the patient’s baseline serum creatinine (sCr, mg/dL) level, dialysis status, New York Heart Association (NYHA) classification, urgency of the procedure, presence of severe lung disease, and type of access (femoral vs. nonfemoral) with a 30-day mortality AUC of 0.66. Since then, other TAVR-specific risk models have been developed to predict 30-day mortality, such as FRANCE-2 (AUC = 0.67)^18^, OBSERVANT (AUC = 0.71)^19^, and CoreValve U.S. (AUC = 0.75)^21^. More recently, a deep learning-based approach was used in^24^ to predict cerebrovascular events (CVEs) post-TAVR using both clinical and imaging data. Their approach resulted in an AUC of 0.79, and they showed that CVEs increased the odds of death by 2.62 and were most likely to occur on the first day post-TAVR. Based on the aforementioned studies, we conclude that our findings are informative since we showed that the use of administrative, preoperative variables with a simple L2 logistic regression model was sufficient to predict in-hospital mortality (with results similar to those of state-of-the-art studies that utilized clinical data, albeit for a 30-day prediction period).

In our estimation, there are three scenarios that can be used to deploy our model in practice. First, practitioners may want to utilize our developed models as is. To assist them in such an instance, we have developed a web app^25^ where they can input the values for the predictor variables based on their patient, and we return both the predicted outcome and the associated probability for survival. Note that the model deployed in the app is trained on the entire NIS data sample based on the recommendation in^26^. Second, the model can be deployed as is using a different snapshot of the NIS database. In this case, we recommend following our five-step approach highlighted in the central illustration. Once satisfactory predictive performance is achieved, the best model should be retrained on all the data prior to model deployment^26^. To assist practitioners in such a scenario, we provide our code in^27^, which they can reuse for their dataset. Third, in large hospital system settings, there may be access to pertinent clinical predictors in addition to NIS variables. Penalized logistic regression (e.g., LASSO, ridge, elastic net, etc.) can be used to model such data. The research question in such a case would be whether clinical data would provide more information when compared to the out-of-network patients whose data would be deleted due to the absence of clinical data.

### Limitations

There are several limitations in this study that need to be highlighted. First, our models were based on the HCUP NIS database. The database was not designed for clinical decision support, and the derivation of clinical information from ICD codes is a limitation since “some nonrelated clinical diagnoses may be omitted and may not represent the true prevalence of risk factors”^3^, and the encoding of such raw health data may be inconsistent across hospitals/providers/time. Second, our models’ predictions were limited to in-hospital mortality. While the post-TAVR survival probability should monotonically decrease over time, the decision to operate on a patient is based on a longer survival time frame and clinical data that were not observed in our study. Third, innovations in TAVR procedures (e.g., an increased prevalence of robot-assisted surgeries) and pre/postprocedural care were not captured in our analyses. Such innovations can significantly improve survival outcomes and deem the historical data used in model training obsolete. Thus, incorporating this domain expertise in training/retraining our statistical/machine learning models would be an important consideration^28^ if they are deployed for decision support.

## Conclusions

Despite the complexity of TAVR procedures and the variability in patient mix, post-TAVR survival and death can be somewhat predicted using only administrative, preoperative data and several standard statistical/machine learning models. Our study illustrates that administrative data can be used to predict and/or risk adjust complex medical procedures, such as TAVR, without the need for frequent retraining.

## Methods

### Data source

The dataset used was acquired from the NIS/HCUP database^5^. The unit of analysis was the discharge record. ICD-9-CM (International Classification of Diseases, Ninth Revision, Clinical Modification) codes 3505 and 3506 were used to identify all patients ≥ 18 years who underwent a TAVR procedure between January 01, 2012, and September 30, 2015. Furthermore, the ICD-10-CM codes 02RF4xx and 02RF3xx were used to identify all patients ≥ 18 years who underwent a TAVR procedure between October 01, 2015, and December 31, 2019.

A total of 54,739 TAVR records were obtained using the aforementioned ICD codes, filtering nonadult patients and removing missing data for age, race, sex, income, elective surgery, and in-hospital mortality. The data were divided into two groups: those who survived the procedure (alive; *n* = 53,626) and those who died during the same hospitalization (deceased; *n* = 1113). For each procedure, the ICD-9-CM (prior to October 01, 2015) or ICD-10-CM (starting from October 01, 2015) was used to identify comorbidities and the TAVR approach (see Supplementary Table S5 for utilized codes).

### Ethical approval

Per the HCUP site^5^, “HCUP databases conform to the definition of a limited data set. A limited data set is healthcare data in which 16 direct identifiers, specified in the Privacy Rule, have been removed. Under HIPAA [the Health Insurance Portability and Accountability Act], review by an institutional review board (IRB) is not required for use of limited data sets.”

### Study design

**Figure 3:**
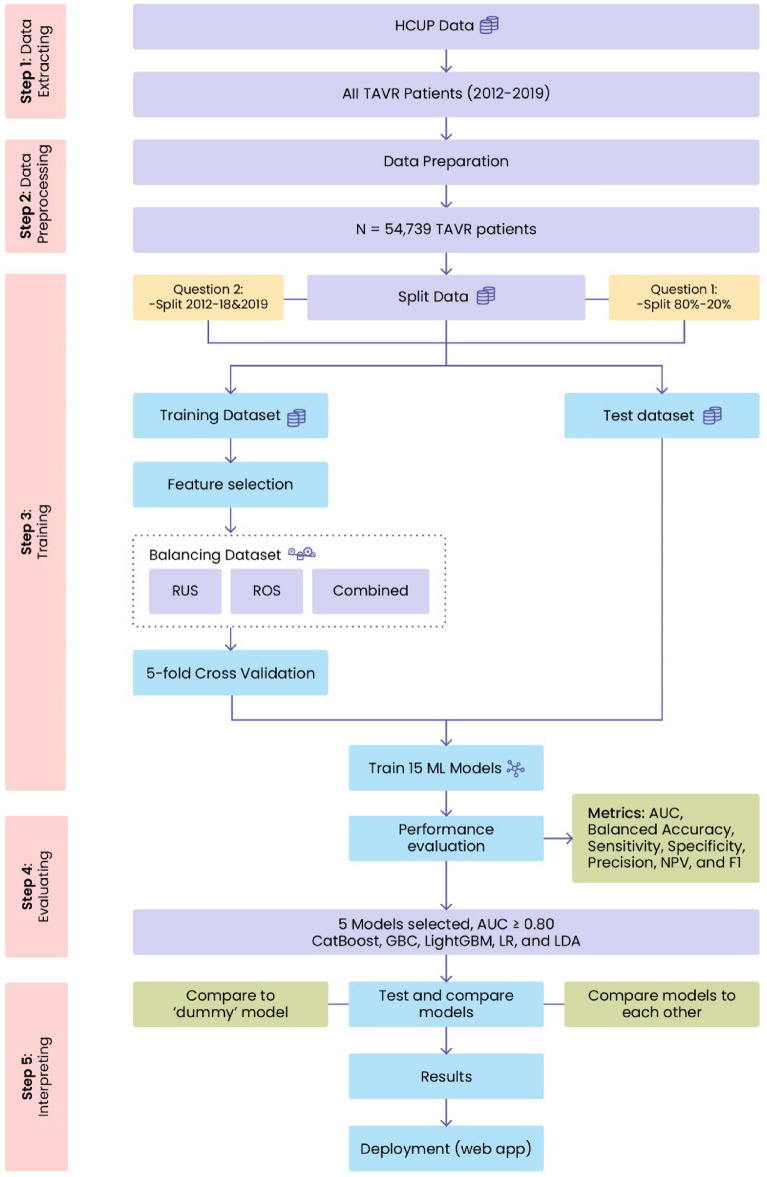
Overview of the modeling workflow of this study.

Figure 3 shows the workflow of this study from data extraction to the use of machine learning techniques to address our two research questions: (a) the utility of NIS preoperative variables alone in predicting TAVR survival and (b) the deployment of such predictive models without frequent retraining. The workflow consisted of five major steps. First, we extracted all TAVR procedures that occurred in 2012–2019 from the NIS database using SAS software (version 9.4, SAS Institute Inc., USA). Second, Python 3.9 was used to prepare the data into a tabular dataset for machine learning, i.e., generate the predictor set that would be used to predict TAVR outcomes. The predictors were divided into patient demographics (age, sex, race, pay information and ZIP code quartile), hospital information (region, bed size, urban/rural/teaching hospital, etc.), and binary indicators of comorbidities. The dataset had 54,739 rows/procedures and 45 columns/variables. The last three steps of training, evaluating, and interpreting the machine learning models were performed separately for each question.

For our first research question, the TAVR dataset was randomly split into an 80% training dataset (*n* = 43,791, with 42,906 alive and 885 deceased individuals) and a 20% test dataset (*n* = 10,948, with 10,720 alive and 228 deceased individuals). On the other hand, for the second research question, the training set contained 40,757 procedures from 2012 to 2018 (39,820 alive and 937 deceased individuals), and the test dataset contained 13,982 procedures from 2019 (13,806 alive and 176 deceased individuals). The training–test split for the second question was 74.5–25.5%.

Given the imbalance between the living and deceased patients in both training samples, we examined the use of random undersampling, random oversampling, and combined resampling to create balanced training datasets^29^ using the imbalance-learn Python library (version 0.9.1). Based on our preliminary analyses, random oversampling resulted in the best prediction performance and hence was used. The resulting training sizes for questions 1 and 2 were 85,812 and 79,640, respectively, each containing an equal number of living and deceased patients.

Similar to^3^, a feature ranking approach was used to examine the top 5/10/20/30/40/all features as inputs in our machine learning models. While the use of external variable/feature selection is not optimal for machine learning models with built-in feature selection^30^, we used this approach to be consistent with^3^ since some of our examined models did not have a built-in feature selection technique (e.g., support vector machines). The external feature selection used was the “classic” method^31^, with a 0.80 threshold, from the PyCaret (version 2. 3.6) Python library^26^.

Using PyCaret, stratified fivefold cross-validation was used to train 15 popular binary classification models for question 1 and 2 training datasets with the aforementioned 5/10/20/30/40/all top features. The 15 models included: (a) traditional statistical models: logistic regression with an L2 penalty (hereafter denoted as LR for conciseness), ridge regression, linear discriminant analysis (LDA), quadratic discriminant analysis, and naïve Bayes; (b) single machine learning classifiers: support vector machines with a linear kernel, k-nearest neighbor classifiers, and decision trees; and (c) ensemble classifiers: gradient boosting classifier (GBC), light gradient boosting machine (LightGBM), CatBoost, Ada Boost classifier, extreme gradient boosting, random forests, and extra trees classifier. Fivefold cross-validation allowed us to select and tune the top five performing models for each question based on the mean AUC. The top five models for the two research questions were the LR, LDA, GBC, LightGBM, and CatBoost models. Both LR^32^ and LDA^32^ are traditional statistical methods/single classifiers. On the other hand, GBC^32^, LightGBM^33^ and CatBoost^34^ are tree-based ensemble methods for binary classification where the predicted class is computed from the mode of predictions from all generated trees. The predictive performance of the top five models was benchmarked against the dummy classifier from PyCaret/scikit-learn, which captures a classifier’s performance when no features/predictors are used. We used the default strategy for the dummy classifier, i.e., prior, which predicted the most frequent class in our training set for all test samples without regard to features. This allowed us to understand the predictive gains obtained from using our administrative features and machine learning models when compared to a dummy classifier. Note that the baselining in a regression problem is somewhat similar since the r^2^ metric captures the improvement in predictive performance compared to just using a dummy model (with the average of the response for prediction irrespective of the values of any potential features).

The five classification models were trained in PyCaret for each of the sets of features and questions. The parameters of the tuned classification models are described in Supplementary Table S6. Furthermore, the dummy model was trained once for each question since it predicted the majority class (i.e., survival post-TAVR for all patients). All of the models were evaluated on the separate (i.e., step 4, not part of training) test sets for questions 1 and 2 using the following performance measures^35,36^: accuracy, AUC, balanced accuracy, sensitivity (recall), specificity, precision (i.e., positive predictive value (PPV)), negative predictive value (NPV), and F1 score. For the sake of conciseness, we did not further describe these models. We refer the reader to the scikit-learn documentation^36^ for a detailed introduction to LR, LDA, and GBC. Similarly, the LightGBM and CatBoost documentation are available from their respective frameworks^37,38^.

In the fifth step of our workflow, we utilized PyCaret to create diagnostic plots of each model’s performance. Due to space limitations, we only show the feature importance plot in this paper.

### Statistical analysis

Following the approach of^3^, a two-tailed t test was used to compare the differences within continuous variables, and chi-square tests were utilized for categorical data. These tests were performed using Minitab software (version 19, Minitab Inc., USA), and *p* < 0.05 was considered statistically significant. The performance of the classification models was assessed using the AUC; however, we also reported other metrics, including accuracy, balanced accuracy, sensitivity/recall, specificity, and precision, as is customary in the literature^29,34^. The training and evaluation of the models were performed using the PyCaret library^26^ in Python.

## Supplementary Information

Supplementary Information.

## Data Availability

The NIS can be purchased from the U.S. Agency for Healthcare Research and Quality (AHRQ). Per their data usage agreement^5^, “I will not redistribute HCUP data by posting on any website or publishing in any other publicly accessible online repository. If a journal or publication requests access to data or analytic files, I will cite restrictions on data sharing in this Data Use Agreement and direct them to AHRQ HCUP (www.hcup-us.ahrq.gov) for more information on accessing HCUP data.”

## References

[1] Aylin P, Bottle A, Majeed A (2007). Use of administrative data or clinical databases as predictors of risk of death in hospital: Comparison of models. BMJ.

[2] Kaafarani HM, Rosen AK (2009). Using administrative data to identify surgical adverse events: An introduction to the patient safety indicators. Am. J. Surg..

[3] Hernandez-Suarez DF (2019). Machine learning prediction models for in-hospital mortality after transcatheter aortic valve replacement. JACC Cardiovasc. Interv..

[4] Groth SS, Habermann EB, Massarweh NN (2020). United States administrative databases and cancer registries for thoracic surgery health services research. Ann. Thorac. Surg..

[5] National Inpatient Sample (NIS). *Healthcare Cost and Utilization Project (HCUP)* (Agency for Healthcare Research and Quality, 2022). 21413206

[6] Zhan C, Miller MR (2003). Administrative data based patient safety research: A critical review. Qual. Saf. Health Care.

[7] Nasr VG, Faraoni D, Valente AM, DiNardo JA (2017). Outcomes and costs of cardiac surgery in adults with congenital heart disease. Pediatr. Cardiol..

[8] Stulberg JJ, Haut ER (2018). Practical guide to surgical data sets: Healthcare cost and utilization project national inpatient sample (NIS). JAMA Surg..

[9] Haut, E. R., Pronovost, P. J. & Schneider, E. B. Limitations of administrative databases. *JAMA* **307**, 2589; author reply 2589–2590 (2012). 10.1001/jama.2012.6626 22735421

[10] Haut ER, Pronovost PJ (2011). Surveillance bias in outcomes reporting. JAMA.

[11] Otto CM (2021). 2020 ACC/AHA guideline for the management of patients with valvular heart disease: Executive summary: A report of the American college of cardiology/American heart association joint committee on clinical practice guidelines. Circulation.

[12] Baladron C, Amat-Santos IJ, San Roman A (2019). Machine learning is no magic: Put a rabbit into the hat before pulling it out. JACC Cardiovasc. Interv..

[13] Modine T, Overtchouk P (2019). Machine learning is no magic: A plea for critical appraisal during periods of hype. JACC Cardiovasc. Interv..

[14] Pollari F, Hitzl W, Claes M, Grossmann I, Fischlein T (2019). Machine learning for making aortic valve interventions complementary and not competitive. JACC Cardiovasc. Interv..

[15] Megahed, F. M., Chen, Y. J., Jones-Farmer, A. & Rigdon, S. *The Variability in Commonly Used Classification Metrics with Class Imbalance*. https://fmegahed.github.io/research/classification/metrics_variability.html (2023).

[16] Edwards FH (2016). Development and validation of a risk prediction model for in-hospital mortality after transcatheter aortic valve replacement. JAMA Cardiol..

[17] Ullah W (2022). Predictors of in-hospital mortality in patients with end-stage renal disease undergoing transcatheter aortic valve replacement: A nationwide inpatient sample database analysis. Cardiovasc. Revasc. Med..

[18] Iung B (2014). Predictive factors of early mortality after transcatheter aortic valve implantation: Individual risk assessment using a simple score. Heart.

[19] Capodanno D (2014). A simple risk tool (the OBSERVANT score) for prediction of 30-day mortality after transcatheter aortic valve replacement. Am. J. Cardiol..

[20] Seiffert M (2014). Development of a risk score for outcome after transcatheter aortic valve implantation. Clin. Res. Cardiol..

[21] Hermiller JB (2016). Predicting early and late mortality after transcatheter aortic valve replacement. J. Am. Coll. Cardiol..

[22] Rudin C (2019). Stop explaining black box machine learning models for high stakes decisions and use interpretable models instead. Nat. Mach. Intell..

[23] Masuadi E (2021). Trends in the usage of statistical software and their associated study designs in health sciences research: A bibliometric analysis. Cureus.

[24] Okuno T (2021). Deep learning-based prediction of early cerebrovascular events after transcatheter aortic valve replacement. Sci. Rep..

[25] Megahed, F. M. *Predicting In-hospital Mortality After TAVR Using Preoperative Variables and Penalized Logistic Regression*. https://huggingface.co/spaces/fmegahed/tavr_project (2022).

[26] Ali, M. *PyCaret: An Open Source, Low-Code Machine Learning Library in Python*. https://www.pycaret.org (2020).

[27] Alhwiti, T. *Predicting In-hospital-mortality After Transcatheter Aortic Valve Replacement*. https://github.com/Alhwiti/Predicting-In-Hospital-Mortality-After-Transcatheter-Aortic-Valve-Replacement (2022). 10.1038/s41598-023-37358-9 PMC10290690 37355688

[28] Ali WB (2021). Implementing machine learning in interventional cardiology: The benefits are worth the trouble. Front. Cardiovasc. Med..

[29] Megahed FM (2021). The class imbalance problem. Nat. Methods.

[30] Kuhn, M. *The Caret Package—Feature Selection Overview*. https://topepo.github.io/caret/feature-selection-overview.html (2019).

[31] Evrimler S (2022). Bladder urothelial carcinoma: Machine learning-based computed tomography radiomics for prediction of histological variant. Acad. Radiol..

[32] Hastie T, Tibshirani R, Friedman JH, Friedman JH (2009). The Elements of Statistical Learning: Data Mining, Inference, and Prediction.

[33] GuolinKe QM (2017). Lightgbm: A highly efficient gradient boosting decision tree. Adv. Neural Inf. Process. Syst..

[34] Prokhorenkova L, Gusev G, Vorobev A, Dorogush AV, Gulin A (2018). CatBoost: Unbiased boosting with categorical features. Adv. Neural Inf. Process. Syst..

[35] Lever J (2016). Classification evaluation: It is important to understand both what a classification metric expresses and what it hides. Nat. Methods.

[36] Varoquaux G (2015). Scikit-learn. GetMobile mob. Comput. Commun..

[37] Python-Package. *Introduction—LightGBM 3.3.2.99 Documentation*. https://lightgbm.readthedocs.io/en/latest/Installation-Guide.html (2022).

[38] Python-Package. *CatBoostClassifier Documentation*. https://catboost.ai/en/docs/concepts/python-reference_catboostclassifier (2022).

