# Supplementary material for: Predicting in-hospital mortality after transcatheter aortic valve replacement using administrative data and machine learning
Source: Sci Rep. 2023 Jun 24;13:10252. doi: 10.1038/s41598-023-37358-9 (PMC10290690; doi:10.1038/s41598-023-37358-9)
Supplement: Supplementary file 1 — Supplementary Information. [file 41598_2023_37358_MOESM1_ESM.docx]

**Supplementary Materials for “Predicting in-hospital mortality after transcatheter aortic valve replacement using administrative data and machine learning”**

# Code repository and web app

Our code is available online at <https://github.com/Alhwiti/Predicting-In-Hospital-Mortality-After-Transcatheter-Aortic-Valve-Replacement>, and our web app can be accessed at <https://huggingface.co/spaces/fmegahed/tavr_project>.

# Online tables and figures

In pages 2-14 (of this document), we provide several online/supplementary tables and figures to supplement the results and discussion in our original paper.

**Online Table 1.**  The demographic, hospital, and comorbidities characteristics of TAVR patients used in question 2.

|  | Overall | | Survived | | Deceased | | P-Value | Training Dataset | | Test Dataset | | P-Value | Time Train | | Time Test | | P-value |
| --- | --- | --- | --- | --- | --- | --- | --- | --- | --- | --- | --- | --- | --- | --- | --- | --- | --- |
| ***Demographic Characteristics*** | N= 54,739 | | 53,626 | | 1,113 | |  | 43,791 | | 10,948 | |  | 39,820 | | 13,982 | |  |
| Age ( years) | 79.65 | ± 8.5 | 79.62 | ± 8.5 | 81 | ± 8.9 | <0.001 | 80 | ± 8.5 | 80 | ± 8.6 | 0.622 | 80 | ± 8.5 | 78 | ± 8.5 | <0.001 |
| Sex ( female) | 25,229 | (46.1) | 24,629 | (45.9) | 600 | (53.9) | <0.001 | 20,215 | (46.2) | 5,014 | (45.8) | 0.494 | 19,061 | (46.8) | 6,188 | (44.1) | <0.001 |
| Race |  |  |  |  |  |  |  |  |  |  |  |  |  |  |  |  |  |
| Asian or Pacific Islander | 702 | (1.9) | 690 | (1.9) | 12 | (1.1) | <0.001 | 547 | (1.2) | 155 | (1.4) | 0.613 | 524 | (1.3) | 178 | (1.3) | 0.02 |
| Black | 2,262 | (4.1) | 2,230 | (4.2) | 32 | (2.8) |  | 1,820 | (4.2) | 442 | (4) |  | 1,698 | (4.2) | 564 | (4) |  |
| Hispanic | 2,487 | (4.5) | 2,419 | (4.5) | 68 | (6.1) |  | 2,000 | (4.6) | 487 | (4.4) |  | 1,869 | (4.6) | 618 | (4.4) |  |
| Native American | 142 | (0.3) | 139 | (0.3) | 3 | (0.3) |  | 116 | (0.3) | 26 | (0.2) |  | 94 | (0.2) | 48 | (0.3) |  |
| White | 47,714 | (87.2) | 46,805 | (87.2) | 909 | (81.7) |  | 38,149 | (87.1) | 9,565 | (87.4) |  | 35,463 | (87) | 12,251 | (87.6) |  |
| Other | 1432 | (2.6) | 1343 | (2.5) | 89 | (7.9) |  | 1,159 | (2.6) | 273 | (2.5) |  | 1,109 | (2.7) | 323 | (2.3) |  |
| Smoker | 20,793 | (38) | 20,537 | (38.3) | 256 | (23) | <0.001 | 16,690 | (38.1) | 4,103 | (37.5) | 0.22 | 15,097 | (37) | 5,696 | (40.7) | <0.001 |
| Dyslipidemia | 38,654 | (70.6) | 38,102 | (71.1) | 552 | (49.6) | <0.001 | 30,954 | (70.7) | 7,700 | (70.3) | 0.468 | 28,276 | (69.4) | 10,378 | (74.2) | <0.001 |
| Atrial fibrillation and flutter | 22,215 | (40.6) | 21,678 | (40.4) | 537 | (48.3) | <0.001 | 17,733 | (40.5) | 4,482 | (40.9) | 0.397 | 16,986 | (41.7) | 5,229 | (37.4) | <0.001 |
| Carotid artery disease | 3,611 | (6.6) | 3,563 | (6.6) | 48 | (4.3) | 0.002 | 2,889 | (6.6) | 722 | (6.6) | 0.993 | 2,777 | (6.8) | 834 | (6) | <0.001 |
| Known CAD | 38,673 | (70.7) | 37,959 | (70.8) | 714 | (64.2) | <0.001 | 30,972 | (70.7) | 7,701 | (70.3) | 0.429 | 29,050 | (71.3) | 9,623 | (68.8) | <0.001 |
| Prior CABG | 9,714 | (17.8) | 9,569 | (17.8) | 145 | (13) | <0.001 | 7,830 | (17.9) | 1,884 | (17.2) | 0.099 | 7,692 | (18.9) | 2,022 | (14.5) | <0.001 |
| Prior ICD | 1,528 | (2.8) | 1,503 | (2.8) | 25 | (2.3) | 0.265 | 1,182 | (2.7) | 346 | (3.2) | 0.01 | 1,192 | (2.9) | 336 | (2.4) | 0.001 |
| Prior MI | 6,980 | (12.8) | 6,861 | (12.8) | 119 | (10.7) | 0.037 | 5,599 | (12.8) | 1,381 | (12.6) | 0.63 | 5,371 | (13.2) | 1,609 | (11.5) | <0.001 |
| Prior PCI | 11,975 | (21.9) | 11,828 | (22.1) | 147 | (13.2) | <0.001 | 9,566 | (21.8) | 2,409 | (22) | 0.718 | 8,854 | (21.7) | 3,121 | (22.3) | 0.14 |
| Prior PPM | 5,351 | (9.8) | 5,278 | (9.8) | 73 | (6.6) | <0.001 | 4,315 | (9.9) | 1,036 | (9.5) | 0.218 | 4,127 | (10.1) | 1,224 | (8.8) | <0.001 |
| Prior TIA/stroke | 76 | (13.9) | 7,482 | (14) | 122 | (11) | 0.004 | 6,071 | (13.9) | 1,533 | (14) | 0.707 | 5,703 | (14) | 1,901 | (13.6) | 0.242 |
| Elective | 44389 | (81) | 43653 | (81.4) | 736 | (66.1) | <0.001 | 35,511 | (81.1) | 888 | (81.1) | 0.999 | 32,645 | (80.1) | 11,744 | (84) | <0.001 |
| Aweekend | 2,247 | (4.1) | 2,165 | (4) | 82 | (7.4) | <0.001 | 1,790 | (4.1) | 457 | (4.2) | 0.683 | 1,826 | (4.5) | 421 | (3) | <0.001 |
| ***Elixhauser comorbidity*** |  |  |  |  |  |  |  |  |  |  |  |  |  |  |  |  |  |
| Anemia | 5,084 | (9.3) | 4,957 | (9.2) | 127 | (11.4) | 0.017 | 4,087 | (9.3) | 997 | (9.1) | 0.466 | 4,406 | (10.8) | 678 | (4.9) | <0.001 |
| Cancer | 1,469 | (2.7) | 1,440 | (2.7) | 29 | (2.6) | 0.871 | 1,176 | (2.7) | 293 | (2.7) | 0.958 | 1,061 | (2.6) | 408 | (2.9) | 0.047 |
| Cardiac arrhythmias | 29,382 | (53.7) | 28,626 | (53.4) | 756 | (67.9) | 0.0001 | 23,498 | (53.7) | 5,884 | (53.7) | 0.872 | 22,561 | (55.4) | 6,821 | (48.8) | <0.001 |
| Chronic kidney disease | 11,978 | (21.9) | 11,613 | (21.7) | 365 | (32.8) | <0.001 | 9,603 | (21.9) | 2,375 | (21.7) | 0.594 | 9,636 | (23.6) | 2,342 | (16.8) | <0.001 |
| Chronic pulmonary disease | 17,549 | (32.1) | 17,113 | (31.9) | 436 | (39.2) | <0.001 | 14,037 | (32.1) | 3,512 | (32.1) | 0.961 | 13,839 | (34) | 3,710 | (26.5) | <0.001 |
| Coagulopathy | 7,706 | (14.1) | 7,353 | (13.7) | 535 | (31.7) | <0.001 | 6,144 | (14) | 1,562 | (14.3) | 0.523 | 6,288 | (15.4) | 1,418 | (10.1) | <0.001 |
| Depression | 4,441 | (8.1) | 4,378 | (8.2) | 63 | (5.7) | 0.002 | 3,535 | (8.1) | 906 | (8.3) | 0.486 | 3,228 | (7.9) | 1,213 | (8.7) | 0.005 |
| Diabetes mellitus | 20,257 | (37) | 19,936 | (37.2) | 321 | (28.8) | <0.001 | 16,258 | (37.1) | 3,999 | (36.5) | 0.246 | 14,944 | (36.7) | 5,313 | (38) | 0.005 |
| Fluid and electrolyte disorder | 9,206 | (16.8) | 8,624 | (16.1) | 582 | (52.3) | <0.001 | 7,397 | (16.9) | 1,809 | (16.5) | 0.357 | 7,367 | (18.1) | 1,839 | (13.2) | <0.001 |
| Heart failure | 40,541 | (74.1) | 39,653 | (73.9) | 888 | (79.8) | <0.001 | 32,427 | (74.1) | 8,114 | (74.1) | 0.89 | 30,325 | (74.4) | 10,216 | (73.1) | 0.002 |
| Hypertension | 48,328 | (88.3) | 47,482 | (88.5) | 846 | (76) | <0.001 | 38,711 | (88.4) | 9,617 | (84.8) | 0.105 | 35,739 | (87.7) | 12,589 | (90) | <0.001 |
| Liver disease | 1888 | (3.5) | 1712 | (3.2) | 176 | (15.8) | <0.001 | 1,504 | (3.4) | 384 | (3.5) | 0.708 | 1,353 | (3.3) | 535 | (3.8) | 0.005 |
| Obesity | 10,359 | (18.9) | 10,218 | (19.1) | 141 | (12.7) | <0.001 | 8,283 | (19.9) | 2,076 | (20) | 0.91 | 7,369 | (18.1) | 2,990 | (21.4) | <0.001 |
| Weight loss | 1893 | (3.5) | 1732 | (3.2) | 161 | (14.5) | <0.001 | 1,517 | (3.5) | 376 | (3.4) | 0.879 | 1,518 | (3.7) | 375 | (2.7) | <0.001 |
| Peripheral vascular disease | 13,220 | (24.2) | 12,859 | (24) | 361 | (32.4) | <0.001 | 10,486 | (24) | 2,734 | (25) | 0.025 | 10,377 | (25.5) | 2,843 | (20.3) | <0.001 |
| Pulmonary circulation disorder | 7,001 | (12.8) | 6,777 | (12.6) | 224 | (20.1) | <0.001 | 5,607 | (12.8) | 1,394 | (12.7) | 0.842 | 4,878 | (12) | 2,123 | (15.2) | <0.001 |
| Valvular disease | 53,831 | (98.3) | 52,760 | (98.4) | 1,071 | (96.2) | <0.001 | 43,048 | (98.3) | 10,783 | (98.5) | 0.165 | 40,113 | (98.4) | 13,718 | (98.1) | 0.014 |
| Drug abuse | 269 | (0.5) | 263 | (0.5) | 6 | (0.6) | 0.818 | 206 | (0.5) | 63 | (0.6) | 0.16 | 196 | (0.5) | 73 | (0.5) | 0.548 |
| Endocarditis | 152 | (0.3) | 139 | (0.3) | 13 | (1.2) | <0.001 | 121 | (0.3) | 31 | (0.3) | 0.903 | 125 | (0.3) | 27 | (0.2) | 0.028 |
| Family history of CAD | 3,778 | (6.9) | 3,735 | (7) | 43 | (3.9) | <0.001 | 3,028 | (6.9) | 750 | (6.9) | 0.813 | 2,668 | (6.6) | 1,110 | (7.9) | <0.001 |
| Median household income (percentiles) |  |  |  |  |  |  |  |  |  |  |  |  |  |  |  |  |  |
| 0 to 25th | 11,696 | (21.4) | 11,453 | (21.4) | 243 | (21.8) | 0.689 | 9,381 | (21.4) | 2,315 | (21.2) | 0.92 | 8,755 | (21.5) | 2,941 | (21) | 0.166 |
| 26th to 50th | 13,684 | (25) | 13,422 | (25) | 262 | (23.6) |  | 10,935 | (25) | 2,749 | (25.1) |  | 10,250 | (25.2) | 3,434 | (24.6) |  |
| 51st to 75th | 14,541 | (26.7) | 14,245 | (26.6) | 296 | (26.6) |  | 11,617 | (26.5) | 2,924 | (26.7) |  | 10,745 | (26.4) | 3,796 | (27.2) |  |
| 76th to 100th | 14,818 | (27.1) | 14,506 | (27.1) | 312 | (28) |  | 11,858 | (27.1) | 2,960 | (27) |  | 11,007 | (27) | 3,811 | (27.3) |  |
| ***Hospital Characteristics*** |  |  |  |  |  |  |  |  |  |  |  |  |  |  |  |  |  |
| Bed Size |  |  |  |  |  |  |  |  |  |  |  |  |  |  |  |  |  |
| Small | 3425 | (6.3) | 3362 | (6.3) | 63 | (5.7) | 0.324 | 2,764 | (6.3) | 661 | (6) | 0.468 | 2,435 | (6) | 990 | (7.1) | <0.001 |
| Medium | 10,816 | (19.8) | 10,611 | (19.8) | 205 | (18.4) |  | 8,671 | (19.8) | 2,145 | (19.6) |  | 7,864 | (19.3) | 2,952 | (21.1) |  |
| Large | 40,498 | (73.9) | 39,653 | (73.9) | 845 | (79.9) |  | 32,356 | (73.9) | 8,142 | (74.4) |  | 30,458 | (74.7) | 10,040 | (71.8) |  |
| Hospital Location |  |  |  |  |  |  |  |  |  |  |  |  |  |  |  |  |  |
| Rural | 536 | (1) | 524 | (1) | 12 | (1.1) | 0.926 | 414 | (1) | 122 | (1.1) | 0.237 | 348 | (0.9) | 188 | (1.3) | <0.001 |
| Urban nonteaching | 4,880 | (8.9) | 4,779 | (8.9) | 101 | (90.7) |  | 3,920 | (9) | 960 | (8.8) |  | 3,715 | (9.1) | 1,165 | (8.3) |  |
| Urban teaching | 49,323 | (90.1) | 48,323 | (90.1) | 1,000 | (89.9) |  | 39,457 | (90.1) | 9,866 | (90.1) |  | 36,694 | (90) | 12,629 | (90.3) |  |
| Hospital Region |  |  |  |  |  |  |  |  |  |  |  |  |  |  |  |  |  |
| Northeast | 13,432 | (24.6) | 13,193 | (24.6) | 239 | (21.5) | <0.001 | 10,813 | (24.7) | 2,619 | (23.9) | 0.146 | 10,099 | (24.8) | 3,333 | (23.8) | <0.001 |
| Midwest | 11,765 | (21.5) | 11,512 | (21.5) | 253 | (22.7) |  | 9,415 | (21.5) | 2,350 | (21.5) |  | 8,606 | (21.1) | 3,159 | (22.6) |  |
| South | 18,742 | (34.2) | 18,304 | (34.1) | 438 | (39.4) |  | 14,998 | (34.3) | 3,744 | (34.2) |  | 14,133 | (34.7) | 4,609 | (33) |  |
| West | 10,800 | (19.7) | 10,617 | (19.8) | 183 | (16.4) |  | 8,565 | (19.6) | 2,235 | (20.4) |  | 7,919 | (19.4) | 2,881 | (20.6) |  |
| Hospital Control |  |  |  |  |  |  |  |  |  |  |  |  |  |  |  |  |  |
| Government Non Federal | 4,206 | (7.7) | 4,100 | (9.5) | 106 | (7.7) | 0.066 | 3,418 | (7.8) | 788 | (7.2) | 0.102 | 3,105 | (7.6) | 1,101 | (7.9) | 0.014 |
| Private Invest Own | 4733 | (8.7) | 4639 | (8.5) | 94 | (8.7) |  | 3,781 | (8.6) | 952 | (8.7) |  | 3,448 | (8.5) | 1,285 | (9.2) |  |
| Private Not Profit | 45,800 | (83.7) | 44,887 | (82) | 913 | (83.7) |  | 36,592 | (83.6) | 9,208 | (84.1) |  | 34,204 | (83.9) | 11,596 | (82.9) |  |
| Hospital Division |  |  |  |  |  |  |  |  |  |  |  |  |  |  |  |  |  |
| East North Central | 8,359 | (15.3) | 8,185 | (15.3) | 174 | (15.6) | <0.001 | 6,686 | (15.3) | 1,673 | (15.3) | 0.071 | 6,215 | (15.3) | 2,144 | (15.3) | <0.001 |
| East South Central | 3,039 | (5.6) | 2,954 | (5.5) | 85 | (7.6) |  | 2,456 | (5.6) | 583 | (5.3) |  | 2,343 | (5.8) | 696 | (5) |  |
| Middle Atlantic | 10,295 | (18.8) | 10,100 | (18.8) | 195 | (17.5) |  | 8,305 | (19) | 1,990 | (18.2) |  | 7,814 | (19.2) | 2,481 | (17.7) |  |
| Mountain | 3,164 | (5.8) | 3,114 | (5.8) | 50 | (4.5) |  | 2,472 | (5.6) | 692 | (6.3) |  | 2,281 | (5.6) | 883 | (6.3) |  |
| New England | 3,137 | (5.7) | 3,093 | (5.8) | 44 | (4) |  | 2,508 | (5.7) | 629 | (5.8) |  | 2,285 | (5.6) | 852 | (6.1) |  |
| Pacific | 7,636 | (14) | 7,503 | (14) | 133 | (12) |  | 6,093 | (13.9) | 1,543 | (14.1) |  | 5,638 | (13.8) | 1,998 | (14.3) |  |
| South Atlantic | 10,805 | (19.7) | 10,563 | (19.7) | 242 | (21.7) |  | 8,670 | (19.8) | 2,135 | (19.5) |  | 8,093 | (19.9) | 2,712 | (19.4) |  |
| West North Central | 3,406 | (6.2) | 3,327 | (6.2) | 79 | (7.1) |  | 2,729 | (6.2) | 677 | (6.2) |  | 2,391 | (5.9) | 1,015 | (7.3) |  |
| West South Central | 4,898 | (9) | 4,787 | (8.9) | 111 | (10) |  | 3,872 | (8.8) | 1,026 | (9.4) |  | 3,697 | (9.1) | 1,201 | (8.6) |  |
| Pay |  |  |  |  |  |  |  |  |  |  |  |  |  |  |  |  |  |
| Medicaid | 712 | (1.3) | 694 | (1.3) | 18 | (1.6) | <0.001 | 579 | (1.3) | 140 | (1.3) | 0.936 | 525 | (1.3) | 194 | (1.4) | <0.001 |
| Medicare | 48902 | (89.3) | 47898 | (89.3) | 1,004 | (90.2) |  | 39,143 | (89.4) | 9,799 | (89.5) |  | 36,656 | (89.9) | 12,286 | (87.9) |  |
| No charge | 18 | (0) | 18 | (0) | - | (0) |  | 16 | (0) | 5 | (0) |  | 11 | (0) | 10 | (0.1) |  |
| Private insurance | 3,983 | (7.3) | 3,910 | (7.3) | 73 | (6.6) |  | 3,187 | (7.3) | 802 | (7.3) |  | 2,837 | (7) | 1,152 | (8.2) |  |
| Self-pay | 245 | (0.4) | 240 | (0.4) | 5 | (0.4) |  | 199 | (0.5) | 49 | (0.4) |  | 188 | (0.5) | 60 | (0.4) |  |
| Other | 879 | (1.6) | 866 | (1.6) | 13 | (1.2) |  | 667 | (1.5) | 153 | (1.4) |  | 540 | (1.3) | 280 | (2) |  |
| Transapical TAVR | 45,078 | (82.4) | 44,308 | (82.6) | 770 | (69.2) | <0.001 | 36,010 | (82.2) | 9,068 | (82.8) | 0.143 | 31,125 | (76.4) | 13,953 | (99.8) | <0.001 |
| Endovascular TAVR | 9,681 | (17.7) | 9,335 | (17.4) | 346 | (31.1) | <0.001 | 7,781 | (17.8) | 1,880 | (17.2) | 0.143 | 9,652 | (23.7) | 29 | (0.2) | <0.001 |

**Online Table 2**. Features ranking for the top 5 ML models (question 1).

| Features | LR | LightGBM | GBC | LDA | CatBoost | Mean | Rank |
| --- | --- | --- | --- | --- | --- | --- | --- |
| Age | 1 | 1 | 5 | 1 | 1 | 1.8 | 1 |
| Fluid And Electrolyte Disorder | 2 | 2 | 1 | 2 | 2 | 1.8 | 2 |
| Liver Disease | 3 | 5 | 2 | 3 | 4 | 3.4 | 3 |
| Hypertension | 4 | 6 | 4 | 4 | 10 | 5.6 | 4 |
| Dyslipidemia | 7 | 7 | 3 | 7 | 5 | 5.8 | 5 |
| Peripheral Vascular Disease | 8 | 3 | 8 | 8 | 3 | 6 | 6 |
| Cardiac Arrhythmias | 6 | 4 | 7 | 6 | 7 | 6 | 7 |
| Coagulopathy | 9 | 10 | 6 | 9 | 9 | 8.6 | 8 |
| Prior PPM | 5 | 11 | 12 | 5 | 11 | 8.8 | 9 |
| Smoker | 10 | 8 | 10 | 10 | 8 | 9.2 | 10 |
| Chronic Kidney Disease | 14 | 9 | 9 | 14 | 12 | 11.6 | 11 |
| Anemia | 11 | 12 | 14 | 11 | 14 | 12.4 | 12 |
| Pulmonary Circulation Disorder | 13 | 13 | 11 | 12 | 15 | 12.8 | 13 |
| Carotid Artery Disease | 15 | 14 | 15 | 15 | 6 | 13 | 14 |
| Cancer | 12 | 15 | 13 | 13 | 13 | 13.2 | 15 |

**Online Table 3**. Top 5 ML models’ performance at the cross validation stage (question 1).

| Research Question | ML Model | # Input Features | AUC | Balanced Accuracy | Sensitivity | Specificity | Accuracy |
| --- | --- | --- | --- | --- | --- | --- | --- |
| Question 1 | LR | All | 0.798 ± 0.017 | 0.731 | 0.692 | 0.772 | 0.77 |
|  |  | 40 | 0.798 ± 0.015 | 0.729 | 0.686 | 0.772 | 0.77 |
|  |  | 30 | 0.799 ± 0.01 | 0.726 | 0.686 | 0.766 | 0.765 |
|  |  | 20 | 0.795 ± 0.014 | 0.72 | 0.675 | 0.765 | 0.763 |
|  |  | 10 | 0.789 ± 0.011 | 0.723 | 0.68 | 0.765 | 0.764 |
|  |  | 5 | 0.748 ± 0.023 | 0.691 | 0.579 | 0.802 | 0.798 |
|  | LightGBM | All | 0.801 ± 0.014 | 0.722 | 0.761 | 0.684 | 0.686 |
|  |  | 40 | 0.804 ± 0.013 | 0.726 | 0.765 | 0.686 | 0.688 |
|  |  | 30 | 0.8 ± 0.12 | 0.725 | 0.776 | 0.674 | 0.676 |
|  |  | 20 | 0.794 ± 0.013 | 0.715 | 0.771 | 0.659 | 0.662 |
|  |  | 10 | 0.787 ± 0.005 | 0.707 | 0.775 | 0.64 | 0.642 |
|  |  | 5 | 0.746 ± 0.015 | 0.668 | 0.73 | 0.606 | 0.609 |
|  | GBC | All | 0.802 ± 0.011 | 0.726 | 0.666 | 0.787 | 0.784 |
|  |  | 40 | 0.798 ± 0.01 | 0.728 | 0.668 | 0.789 | 0.786 |
|  |  | 30 | 0.799 ± 0.01 | 0.728 | 0.685 | 0.771 | 0.77 |
|  |  | 20 | 0.797 ± 0.015 | 0.727 | 0.686 | 0.769 | 0.767 |
|  |  | 10 | 0.792 ± 0.005 | 0.719 | 0.677 | 0.762 | 0.76 |
|  |  | 5 | 0.745 ± 0.012 | 0.691 | 0.597 | 0.785 | 0.782 |
|  | LDA | All | 0.798 ± 0.015 | 0.73 | 0.673 | 0.786 | 0.783 |
|  |  | 40 | 0.799 ± 0.014 | 0.732 | 0.679 | 0.785 | 0.783 |
|  |  | 30 | 0.8 ± 0.01 | 0.725 | 0.669 | 0.781 | 0.779 |
|  |  | 20 | 0.795 ± 0.013 | 0.724 | 0.668 | 0.78 | 0.778 |
|  |  | 10 | 0.789 ± 0.011 | 0.722 | 0.663 | 0.78 | 0.778 |
|  |  | 5 | 0.745 ± 0.022 | 0.691 | 0.554 | 0.828 | 0.822 |
|  | CatBoost | All | 0.794 ± 0.019 | 0.727 | 0.654 | 0.8 | 0.797 |
|  |  | 40 | 0.796 ± 0.02 | 0.726 | 0.651 | 0.801 | 0.798 |
|  |  | 30 | 0.798 ± 0.025 | 0.728 | 0.669 | 0.787 | 0.784 |
|  |  | 20 | 0.789 ± 0.012 | 0.72 | 0.669 | 0.771 | 0.768 |
|  |  | 10 | 0.786 ± 0.008 | 0.718 | 0.676 | 0.76 | 0.759 |
|  |  | 5 | 0.741 ± 0.012 | 0.689 | 0.597 | 0.782 | 0.778 |
| Question 2 | LR | All | 0.78 ± 0.025 | 0.713 | 0.679 | 0.747 | 0.746 |
|  |  | 40 | 0.78 ± 0.026 | 0.713 | 0.68 | 0.747 | 0.745 |
|  |  | 30 | 0.78 ± 0.034 | 0.702 | 0.685 | 0.718 | 0.717 |
|  |  | 20 | 0.784 ± 0.042 | 0.706 | 0.693 | 0.719 | 0.718 |
|  |  | 10 | 0.777 ± 0.039 | 0.708 | 0.668 | 0.748 | 0.747 |
|  |  | 5 | 0.734 ± 0.047 | 0.683 | 0.575 | 0.79 | 0.785 |
|  | LightGBM | All | 0.791 ± 0.025 | 0.712 | 0.774 | 0.651 | 0.654 |
|  |  | 40 | 0.785 ± 0.025 | 0.711 | 0.773 | 0.649 | 0.652 |
|  |  | 30 | 0.774 ± 0.038 | 0.687 | 0.768 | 0.606 | 0.61 |
|  |  | 20 | 0.783 ± 0.038 | 0.691 | 0.778 | 0.604 | 0.608 |
|  |  | 10 | 0.778 ± 0.034 | 0.701 | 0.782 | 0.62 | 0.624 |
|  |  | 5 | 0.738 ± 0.047 | 0.669 | 0.754 | 0.584 | 0.588 |
|  | GBC | All | 0.785 ± 0.02 | 0.715 | 0.663 | 0.767 | 0.764 |
|  |  | 40 | 0.783 ± 0.022 | 0.717 | 0.669 | 0.764 | 0.762 |
|  |  | 30 | 0.774 ± 0.039 | 0.697 | 0.681 | 0.713 | 0.712 |
|  |  | 20 | 0.781 ± 0.035 | 0.705 | 0.67 | 0.739 | 0.738 |
|  |  | 10 | 0.775 ± 0.032 | 0.701 | 0.636 | 0.766 | 0.763 |
|  |  | 5 | 0.739 ± 0.035 | 0.684 | 0.583 | 0.784 | 0.78 |
|  | LDA | All | 0.779 ± 0.027 | 0.717 | 0.677 | 0.757 | 0.755 |
|  |  | 40 | 0.78 ± 0.026 | 0.719 | 0.674 | 0.764 | 0.762 |
|  |  | 30 | 0.778 ± 0.035 | 0.706 | 0.674 | 0.739 | 0.737 |
|  |  | 20 | 0.784 ± 0.043 | 0.707 | 0.672 | 0.741 | 0.74 |
|  |  | 10 | 0.774 ± 0.041 | 0.709 | 0.658 | 0.76 | 0.758 |
|  |  | 5 | 0.729 ± 0.048 | 0.683 | 0.549 | 0.817 | 0.81 |
|  | CatBoost | All | 0.778 ± 0.013 | 0.712 | 0.65 | 0.773 | 0.771 |
|  |  | 40 | 0.775 ± 0.017 | 0.706 | 0.634 | 0.779 | 0.775 |
|  |  | 30 | 0.76 ± 0.031 | 0.688 | 0.642 | 0.734 | 0.732 |
|  |  | 20 | 0.777 ± 0.033 | 0.7 | 0.682 | 0.718 | 0.717 |
|  |  | 10 | 0.771 ± 0.009 | 0.7 | 0.658 | 0.742 | 0.74 |
|  |  | 5 | 0.725 ± 0.026 | 0.683 | 0.56 | 0.805 | 0.8 |

**Online Table 4**. Top models with postoperative predictors (to highlight that Suarez et al.’s predictive performance is driven by postoperative features).

| Classifier | AUC | Sensitivity | Specificity | Accuracy | Recall | Prec. | Balanced Accuracy |
| --- | --- | --- | --- | --- | --- | --- | --- |
| LightGBM | 93.55 | 84.65 | 87.74 | 0.8768 | 0.8465 | 0.1281 | 0.862 |
| GBC | 93.22 | 82.46 | 90.57 | 0.904 | 0.8246 | 0.1568 | 0.8651 |
| CatBoost | 91.61 | 76.32 | 90.88 | 0.9057 | 0.7632 | 0.151 | 0.836 |
| LR | 93.05 | 81.14 | 90.6 | 0.904 | 0.8114 | 0.1551 | 0.8587 |
| LDA | 92.62 | 77.19 | 91.89 | 0.9159 | 0.7719 | 0.1684 | 0.8454 |

**Online Table 5**. International classification of diseases, ICD – 9 CM and ICD – 10 codes used to identify baseline comorbidities, and procedures

| Variables | ICD – 9 | ICD – 10 |
| --- | --- | --- |
| *Comorbidities* | | |
| Atrial fibrillation and flutter | 427.31, 427.32 | I48.x |
| Carotid artery disease | 433.10 | I65.2x |
| Dyslipidemia | 272.0 - 272.4 | E78.0 - E78.5 |
| Known CAD | 412, 414.x, 440.3x, V45.81, V45.82 | I25.x, I70.3x - I70.9x, Z95.1, Z95.5, Z95.820, Z98.6x |
| Prior CABG | V45.81 | Z95.1 |
| Prior ICD | V45.02 | Z95.810 |
| Prior MI | 412 | I25.2 |
| Prior PCI | V45.82 | Z95.5, Z95.820, Z98.6x |
| Prior PPM | V45.01 | Z95.0 |
| Prior TIA/stroke | 438.x, V12.54 | I69.3x, Z86.73 |
| Smoker | 305.1, V15.82 | F17.x, T65.2x, Z57.31, Z71.6, Z72.0, Z77.22, Z87.891 |
| Anemia | 280.x - 281.x, 285.2x - 285.9 | D50.0, D50.8, D50.9, D51.x - D53.x |
| Cancer | 140.x - 172.x, 174.x - 199.x | C00.x - C43.x, C45.x - C80.x, C97.x |
| Cardiac arrhythmias | 426.0 - 426.11, 426.13 - 426.53, 426.6 - 426.9, 427.0 - 427.4, 427.6 - 427.9, 785.0, V45.0, V53.3 | I44.1 - I44.3, I45.6, I45.9, I47.x - I49.x, R00.0, R00.1, R00.8, T82.1, Z45.0, Z95.0 |
| Chronic kidney disease | 403.01, 403.11, 403.91, 404.02, 404.03, 404.12, 404.13, 404.92, 404.93, 585.x, 586.x, 588.0, V42.0, V45.1x, V56.x | I12.0, I13.1, N18.x, NI9, N25.0, Z49.0 - Z49.2, Z94.0, Z99.2 |
| Chronic pulmonary disease | 416.8, 416.9, 490.x - 505.x, 506.4, 508.1, 508.8 | I27.8, 127.9, J40.x - J47.x, J60.x - J67.x, J68.4, J70.1, J70.3 |
| Coagulopathy | 286.x, 287.1, 287.3 -2 87.5 | D65 - D68.x, D69.1, D69.3 - D69.6 |
| Diabetes mellitus | 250.x, 775.1 | E08.x, E09.x, E10.x, E11.x, E12.x, E13.x, E14.x, P70.2 |
| Fluid and electrolyte disorder | 276.x, 253.6 | E22.2, E86.x, E87.x |
| Heart failure | 398.91, 402.01, 402.11, 402.91, 404.01, 404.03, 404.11, 404.13, 404.91, 404.93, 428.x, 425.4 - 425.9 | I09.9, I11.0, I13.0, I13.2, I25.5, I42.0, I42.5-I42.9, I43.x, I50.x, P29.0 |
| Hypertension | 401.x - 405.x, 437.2 | I10.x - I16.x, 167.4 |
| Liver disease | 070.22, 070.23, 070.32, 070.33, 070.44, 070.54, 070.6, 070.9, 456.0 - 456.2, 570.X, 571.x, 572.2 - 572.8, 573.3, 573.4, 573.8, 573.9, V42.7 | B18.x, I85.x, I86.4, I98.2, K70.x, K71.1, K71.3 - K71.5, K71.7, K72.x- K74.x, K76.0, K76.2 - K76.9, K77, Z94.4 |
| Obesity | 278.0, 783.1, V85.3x, V85.4x | E66.x, Z68.3x, Z68.4x, R63.5 |
| Peripheral vascular disease | 093.0, 437.3, 440.x, 441.x, 442.x, 443.1 - 443.9, 447.1, 557.1, 557.9, V43.4 | I70.x, I71.x, I73.1, I73.8, I73.9, I77.1, I79.0, I79.2, K55.1, K55.8, K55.9, Z95.8, Z95.9 |
| Pulmonary circulation disorder | 415.x, 416.x, 417.0, 417.8, 417.9 | I26.x, I27.x, I28.0, I28.8, I28.9 |
| Valvular disease | 093.2x, 394.x - 397.x, 424.x, 746.3 - 746.6, V42.2, V43.3 | A52.0, I05.x - I08.x, I09.1, I09.8, I34.x - I39.x, Q23.0 - Q23.3, Z95.2 - Z95.4 |
| Depression | 296.2, 296.3, 296.5, 300.4, 301.12, 309.x, 311 | F20.4, F31.3 - F31.5, F32.x, F33.x, F34.1, F41.2, F43.2 |
| Endocarditis | 421.0, 421.9, 424.9x | I33.x, I38, I39 |
| Family History of CAD | V17.3, V17.4x | Z82.4x |
| Weight loss | 260.x - 263.x, 783.2x, 799.4 | E40.x - E46.x, R63.4, R64 |
| Drug abuse | 292.x, 304.x, 305.2 - 305.9, V65.43 | F11.x - F16.x, F18.x, F19.x, Z71.5, Z72.2 |
| *TAVR Approach* | | |
| Endovascular TAVR | 35.05 | 02RF4xx |
| Transapical TAVR | 35.06 | 02RF3xx |

**Online Table 6**. Machine learning classifier models tuning parameters.

| Machine Learning Models | Tuning Parameters |
| --- | --- |
| Logistic Regression | 'C': 7.573, 'class_weight': {}, 'dual': False, 'fit_intercept': True, 'intercept_scaling': 1, 'l1_ratio': None, 'max_iter': 1000, 'multi_class': 'auto', 'n_jobs': None, 'penalty': 'l2', 'random_state': 2022, 'solver': 'lbfgs', 'tol': 0.0001, 'verbose': 0, 'warm_start': False |
| Light Gradient Boosted Machine Classifier | boosting_type='gbdt', class_weight={0: 6, 1: 9}, colsample_bytree=1.0, importance_type='split', learning_rate=0.1, max_depth=3, min_child_samples=20, min_child_weight=0.001, min_split_gain=0.0, n_estimators=100, n_jobs=-1, num_leaves=80, objective=None, random_state=2022, reg_alpha=0.0, reg_lambda=0.0, silent='warn', subsample=1.0, subsample_for_bin=200000, subsample_freq=0 |
| Gradient Boosting Classifier | ccp_alpha=0.0, criterion='friedman_mse', init=None, learning_rate=0.1, loss='deviance', max_depth=3, max_features=None, max_leaf_nodes=None, min_impurity_decrease=0.0, min_impurity_split=None, min_samples_leaf=1, min_samples_split=2, min_weight_fraction_leaf=0.0, n_estimators=200, n_iter_no_change=None, presort='deprecated', subsample=1.0, tol=0.0001, validation_fraction=0.1,warm_start=False |
| Linear Discriminant Analysis | n_components': None, 'priors': None, 'shrinkage': 0.1, 'solver': 'lsqr', 'store_covariance': False, 'tol': 0.0001 |
| Catboost Classifier | nan_mode': 'Min', 'eval_metric': 'Logloss', 'iterations': 180, 'sampling_frequency': 'PerTree', 'leaf_estimation_method': 'Newton', 'grow_policy': 'SymmetricTree', 'penalties_coefficient': 1, 'boosting_type': 'Plain', 'model_shrink_mode': 'Constant', 'feature_border_type': 'GreedyLogSum', 'bayesian_matrix_reg': 0.10000000149011612, 'force_unit_auto_pair_weights': False, 'l2_leaf_reg': 7, 'random_strength': 0.10000000149011612, 'rsm': 1, 'boost_from_average': False, 'model_size_reg': 0.5, 'pool_metainfo_options': {'tags': {}}, 'subsample': 0.800000011920929, 'use_best_model': False, 'class_names': [0, 1], 'random_seed': 2022, 'depth': 2, 'posterior_sampling': False, 'border_count': 254, 'classes_count': 0, 'auto_class_weights': 'None', 'sparse_features_conflict_fraction': 0, 'leaf_estimation_backtracking': 'AnyImprovement', 'best_model_min_trees': 1, 'model_shrink_rate': 0, 'min_data_in_leaf': 1, 'loss_function': 'Logloss', 'learning_rate': 0.30000001192092896, 'score_function': 'Cosine', 'task_type': 'CPU', 'leaf_estimation_iterations': 10, 'bootstrap_type': 'MVS', 'max_leaves': 4 |

**Online Figure 1**. Performance of models by the number of inputted variables (question 2).


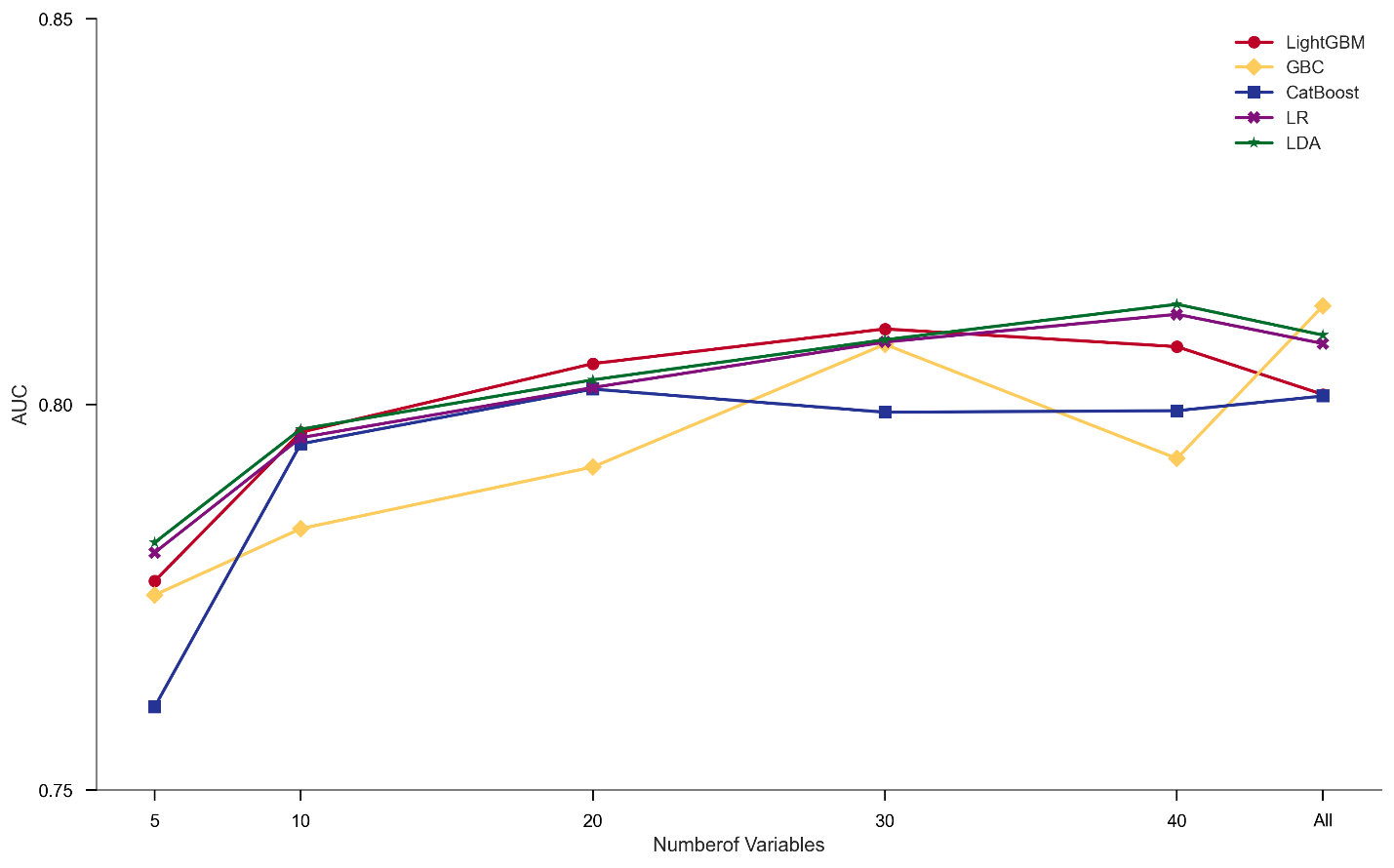


**Online Figure 2**. The area under the ROC curve for model comparison (question 1).

**
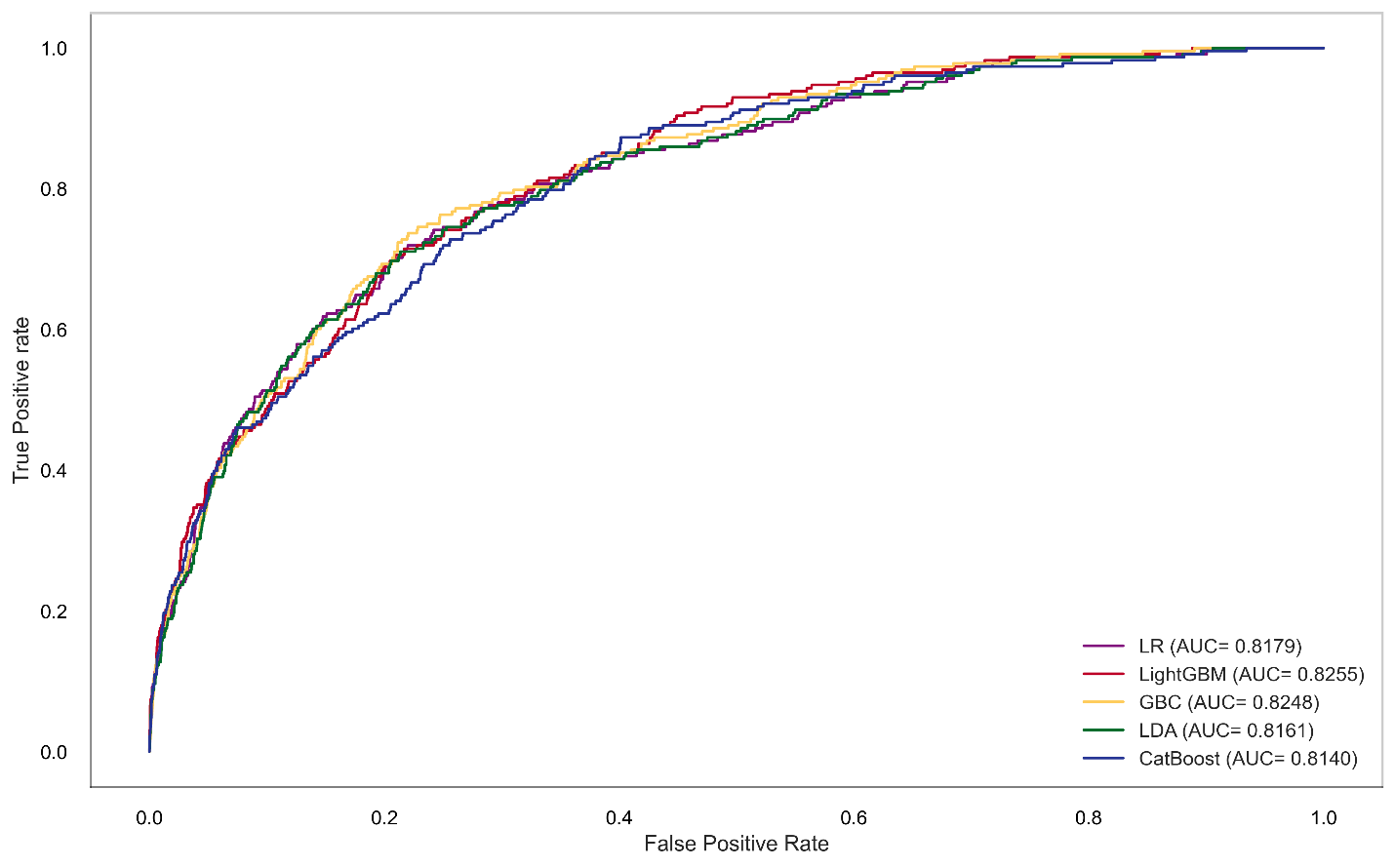
**

**Online Figure 3**. The area under the ROC curve for model comparison (question 2).**
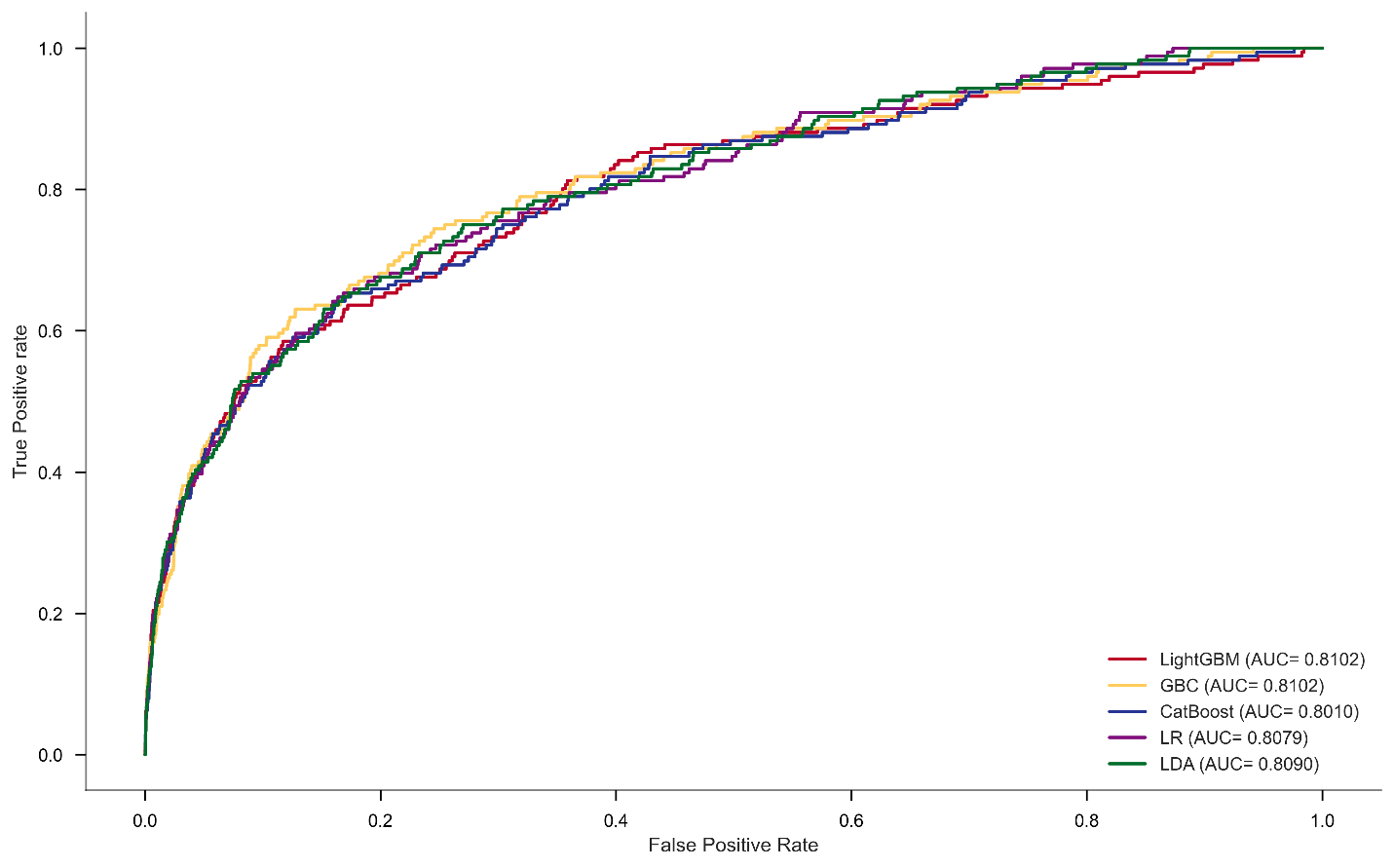
**

**Online Figure 4**. Ranked feature importance for each of the top five models based on mean ranking for research question 2. **
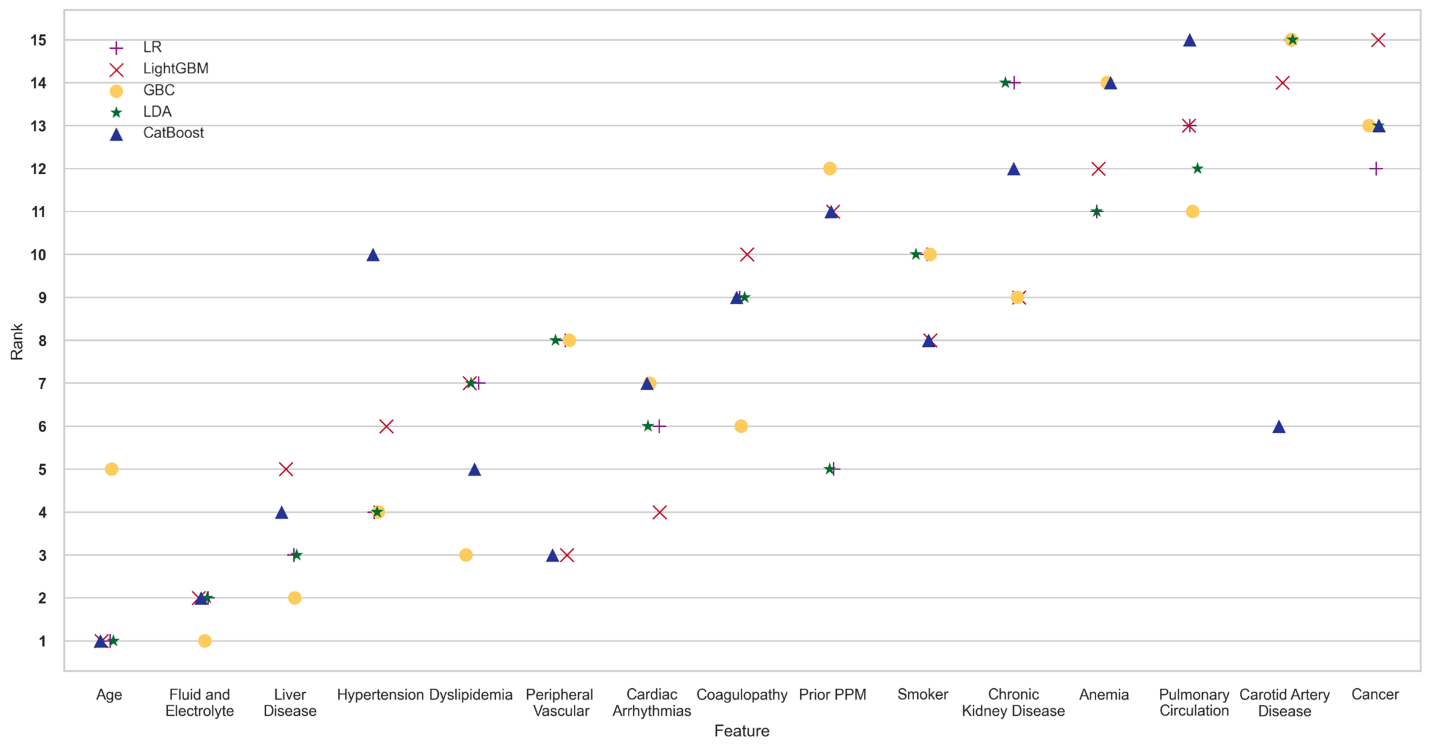
**
